# Advancing a causal role of type 2 diabetes and its components in developing macro‐ and microvascular complications via genetic studies

**DOI:** 10.1111/dme.14982

**Published:** 2022-10-31

**Authors:** Altayeb Ahmed, Naveed Sattar, Hanieh Yaghootkar

**Affiliations:** ^1^ Department of Life Sciences, Centre for Inflammation Research and Translational Medicine Brunel University London London UK; ^2^ School of Cardiovascular and Metabolic Health University of Glasgow Glasgow UK

**Keywords:** genetics of type 2 diabetes, macrovascular disease, mendelian randomization, microvascular disease

## Abstract

The role of diabetes in developing microvascular and macrovascular complications has been subject to extensive research. Despite multiple observational and genetic studies, the causal inference of diabetes (and associated risk factors) on those complications remains incomplete. In this review, we focused on type 2 diabetes, as the major form of diabetes, and investigated the evidence of causality provided by observational and genetic studies. We found that genetic studies based on Mendelian randomization provided consistent evidence of causal inference of type 2 diabetes on macrovascular complications; however, the evidence for causal inference on microvascular complications has been somewhat limited. We also noted high BMI could be causal for several diabetes complications, notable given high BMI is commonly upstream of type 2 diabetes and the recent calls to target weight loss more aggressively. We emphasize the need for further studies to identify type 2 diabetes components that mostly drive the risk of those complications. Even so, the genetic evidence summarized broadly concurs with the need for a multifactorial risk reduction approach in type 2 diabetes, including addressing excess adiposity.

## 1 INTRODUCTION

Diabetes is a complex metabolic disorder characterized by high glucose levels in the blood (hyperglycaemia) due to insufficient insulin secretion and/or resistance to insulin's action.^1^ Diabetes begets disease within different organs, including the eyes, kidneys, heart and blood vessels. These vascular complications are one of the main factors behind the significant morbidity (mainly microvascular complications, e.g. neuropathy, nephropathy and retinopathy) and mortality (mainly macrovascular complications, e.g. coronary heart disease and stroke) associated with the disease. The approach to managing macrovascular and microvascular complications formerly focused heavily on glycaemic control but greater emphasis over the years has been placed on blood pressure, lipids and more recently better weight management, as well as use of novel diabetes agents which lower vascular risks.

Even though most published statements and guidelines encourage tight glycaemic control as a way of lowering the risk of micro‐ and macrovascular complications, the results from trials have shown modest benefits. For example, a recent meta‐analysis of intensive glucose lowering trials reported a reduction in the risk of kidney events by 20% and by 13% for eye events, but the risk was not reduced for nerve events.^2^ An earlier meta‐analysis of the same trials identified intensive glycaemic control resulted in 17% reduction in risk of non‐fatal myocardial infarction and 15% reduction in risk of coronary heart disease but had no significant effect on events of stroke or all‐cause mortality.^3^ While it must be remembered these trials were relatively short term, these modest findings raise the question of what factors other than hyperglycaemia per se contribute to developing those complications among people with diabetes. More recently, SGLT2 inhibitors^4^ and GLP‐1 receptor agonists^5^ have been shown to lower cardiovascular and cardiorenal outcomes in people with type 2 diabetes by levels that cannot be explained by reduction in glucose per se.

**FIGURE 1:**
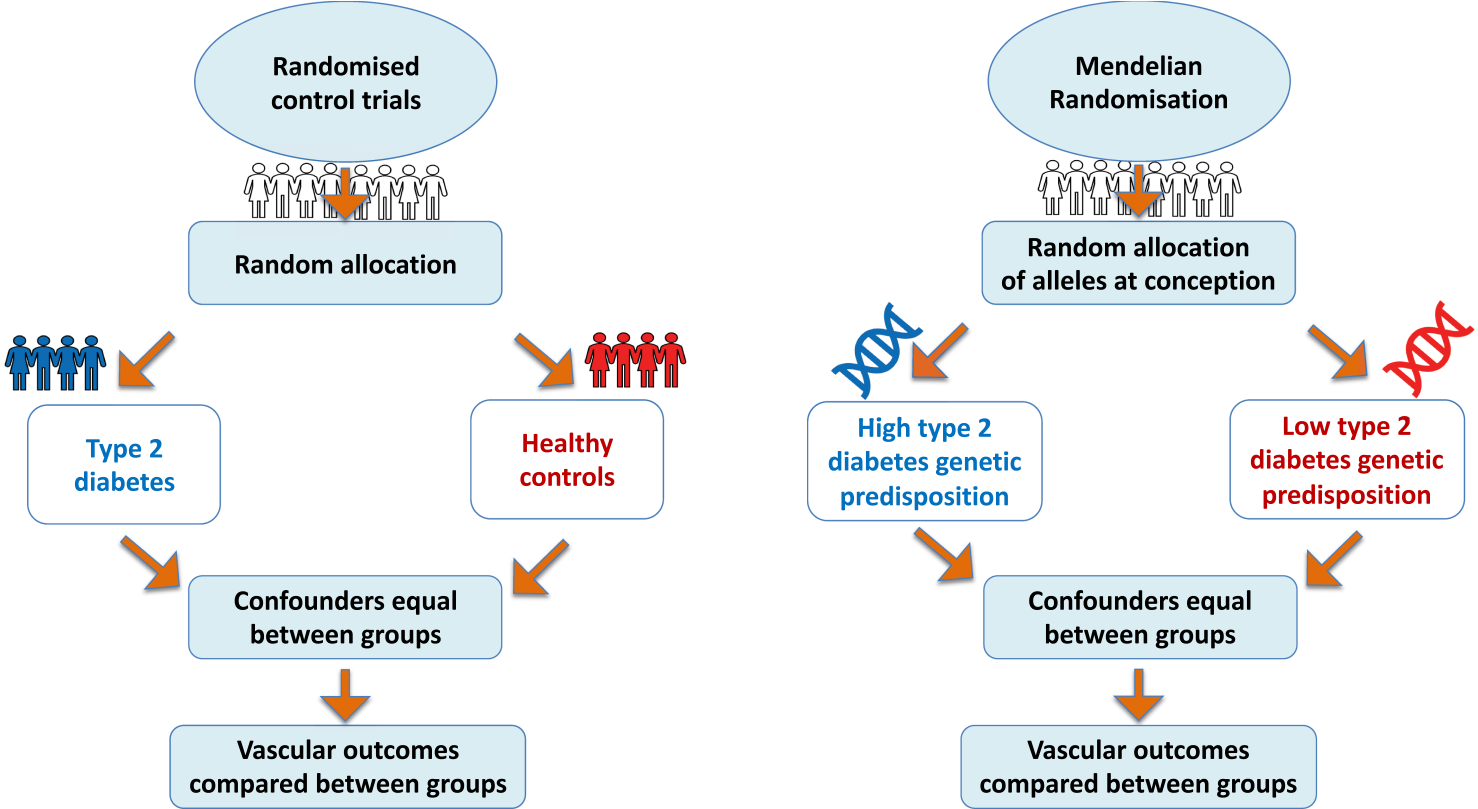
The analogy between Mendelian randomization and Randomized Controlled Trials (RCT). In an RCT, participants are randomly assigned to either treatment or control group and receive a different treatment or management protocol. Consequently, reverse causation and bias are significantly reduced as randomization and group assignment is done at the start of the study. In Mendelian randomization, participants are grouped according to their genetic risk profile for the exposure of interest. For example, to investigate whether type 2 diabetes is causally associated with vascular complications, individuals are randomized based on their genetically defined type 2 diabetes liability. The random inheritance of genetic variants from each parent independent of the outcome, environment and lifestyle factors, reduces the chance of reverse causation and confounding factors.

In this review, we focus on type 2 diabetes as the dominant type of the disease which accounts for 90% of all types. We appraise the evidence gained from studies employing a Mendelian randomization strategy (Figure 1) to answer whether type 2 diabetes is causally associated with microvascular and macrovascular complications, and if so, which component(s) of type 2 diabetes drives this risk.

## 2 DIABETES COMPLICATIONS

Microvascular complications refer to those long‐term complications that affect small blood vessels. Diabetic retinopathy is the most common diabetes‐associated microvascular complication and the leading cause of visual loss among people with diabetes.^6^ Several studies have investigated the pathophysiological role of hyperglycaemia in developing diabetic retinopathy.^7^, ^8^ However, a clear mechanism is yet to be established.^9^ Diabetic nephropathy (either persistent albuminuria or evidence of low eGFR, or often both) develops in 40% of people with all types of diabetes and is the leading cause of chronic kidney disease (eGFR below the threshold of 60 ml/min per 1.73 m^2^)^10^ among those affected with diabetes worldwide.^11^ The aetiology of diabetic nephropathy is not clearly understood. Diabetic neuropathy includes a large spectrum of neuropathic syndromes, including sensory, motor and autonomic peripheral neuropathy. Diabetic polyneuropathy is the most common type of diabetes‐associated neuropathies and affects around 50% of people with different types of diabetes.^12^

Macrovascular complications refer to damage in the body's large blood vessels. Previous studies have found two‐ to three fold increased risk of coronary heart disease among those with type 2 diabetes.^13^ Insulin resistance and obesity play a role in developing coronary heart disease.^14^ However, a clear causal association is yet to be established, with recent studies proposing an effect of other factors such as low socio‐economic status that could partially mediate the link between insulin resistance, obesity and cardiovascular diseases.^15^ Stroke is a major cerebrovascular complication that is associated with type 2 diabetes. People diagnosed with type 2 diabetes are highly susceptible to a cerebral small vessel disease.^14^ The INTERSTROKE study, which was conducted across 22 countries, reported a 35% higher risk for stroke among those with a previous history of diabetes.^16^

## 3 LIMITATIONS OF OBSERVATIONAL STUDIES IN ESTABLISHING A CAUSAL LINK BETWEEN TYPE 2 DIABETES AND MICRO/MACROVASCULAR COMPLICATIONS

Observational studies have already established a clear link between type 2 diabetes and various micro‐ and macrovascular complications. However, determining whether there is a causal relationship between type 2 diabetes and vascular complications has been challenging. Lack of randomization, susceptibility to bias (e.g. measurement error, small sample size), the existence of confounding factors (e.g. obesity may independently influence both the risk of type 2 diabetes and vascular complications) and reverse causation (e.g. the development of vascular disease could precede and accelerate the development of type 2 diabetes) make observational studies often less capable of establishing a causal link between type 2 diabetes (or its components) and its associated vascular complications.^17^

Randomized controlled trials (RCTs) remain the gold standard for establishing a causal association. However, they are often difficult to perform, costly and methodologically difficult to address the question of which aspects of type 2 diabetes causes vascular complications, as few interventions influence only one risk factor. Furthermore, many treatments for hyperglycaemia influence multiple pathways so it is near impossible to dissect out what aspect of a drug therapy lowers risk. This is particularly true for the newer SGLT2i and GLP‐1RAs.^18^ Application of a genetic analogue, called Mendelian randomization, for the RCT (Figure 1) can help overcome many shortcomings of observational studies in a safe, reliable and, often inexpensive manner.^19^ This method is for investigating the existence of a causal relationship between environmental, lifestyle or disease exposures (e.g. type 2 diabetes) and an outcome (e.g. vascular complications).

## 4 THE PRINCIPLES OF MENDELIAN RANDOMIZATION

**FIGURE 2:**
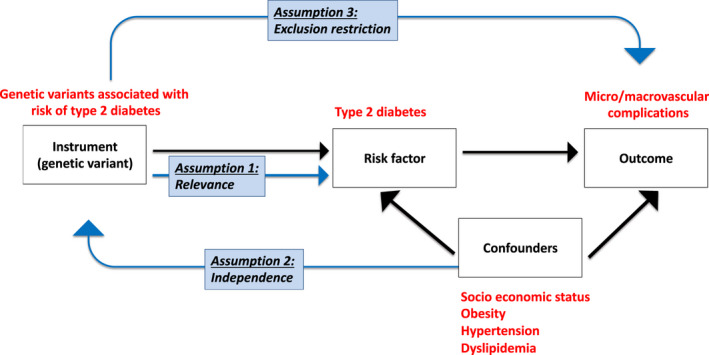
The assumptions of the Mendelian randomization method. First, the relevance assumption implies that instrumental variables must be associated with the exposure of interest. Second, the independence assumption states that there has to be no shared common cause between the instrumental variants and confounding factors. Third, the exclusion restriction assumption implies that the instrument variables do not affect the outcome except through the risk factor of interest.

Mendelian randomization is a statistical method that uses genetic variants (instrumental variables) as proxies for environmental and lifestyle exposure to find evidence of causal inference between a potentially modifiable risk factor and a disease. The method is based on Mendel's law of independent assortment, where genes are inherited randomly from parents to offspring.^20^, ^21^ Valid instrumental variables are fundamental for the success of a Mendelian randomization study. The instrument's validity is satisfied by three assumptions that must be evaluated before using the genetic instrument (Figure 2). First, the relevance assumption implies that instrumental variables must be associated with the exposure of interest. Second, the independence assumption states that there has to be no shared common cause between the instrumental variants and confounding factors. Third, the exclusion restriction assumption implies that the instrument variables do not affect the outcome except through the risk of interest.^22^

As shown in Figure 1, Mendelian randomization is designed similarly to RCTs. In an RCT, participants are randomly assigned to either treatment or control group and receive a different treatment or management protocol. Consequently, reverse causation and bias are eliminated as randomization is done before the study. In Mendelian randomization, participants are grouped according to their genetic risk profile for the exposure of interest. The random inheritance of genetic variants from each parent independent of the outcome, environment and lifestyle factors, reduces the chance of reverse causation and confounding factors.^23^ Using statistical analysis based on genetic variants as an instrument, in a Mendelian randomization design, eliminates the interference of known and unknown confounders and the possibility of reverse causality because genes are inherited randomly and remain nonmodifiable during the course of life.^24^

**FIGURE 3:**
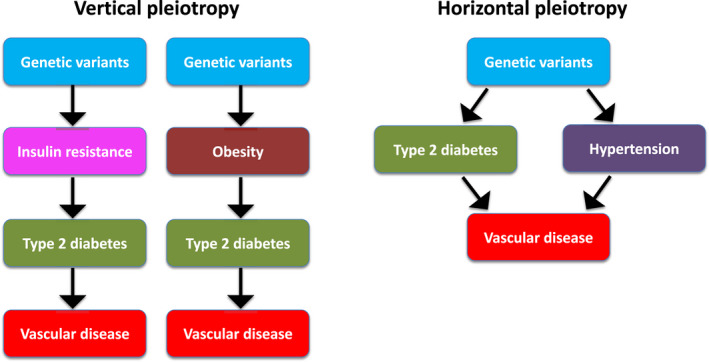
Pleiotropy in Mendelian randomization. A ‘vertical pleiotropy’ occurs if the genetic instrument associates with other traits downstream of the exposure of interest. A ‘horizontal pleiotropy’ occurs when the genetic instrument is associated with traits that are on other independent pathways.

Although Mendelian randomization is the best alternative for RCT in estimating causal relationships, some cautions are needed in interpreting the results when applying this method.^25^ First, the major issue is a phenomenon known as ‘pleiotropy’ where the genetic instrument is associated with other traits (potential exposures or confounders) (Figure 3). Second, the weak instrument can bias the findings towards false negative causal association. Therefore, it is important to use a genetic instrument that is robustly and strongly associated with the exposure of interest. Third, since the discovery of majority of genetic instruments is performed in Europeans only, the generalizability of Mendelian randomization results to other ethnic groups is limited. Fourth, Mendelian randomization estimates the effect of a risk factor over a lifetime and cannot estimate the effect of an intervention at a specific age.

## 5 EVIDENCE FROM MENDELIAN RANDOMIZATION STUDIES FOR A CAUSAL ASSOCIATION BETWEEN TYPE 2 DIABETES AND MICRO/MACROVASCULAR COMPLICATIONS

**FIGURE 4:**
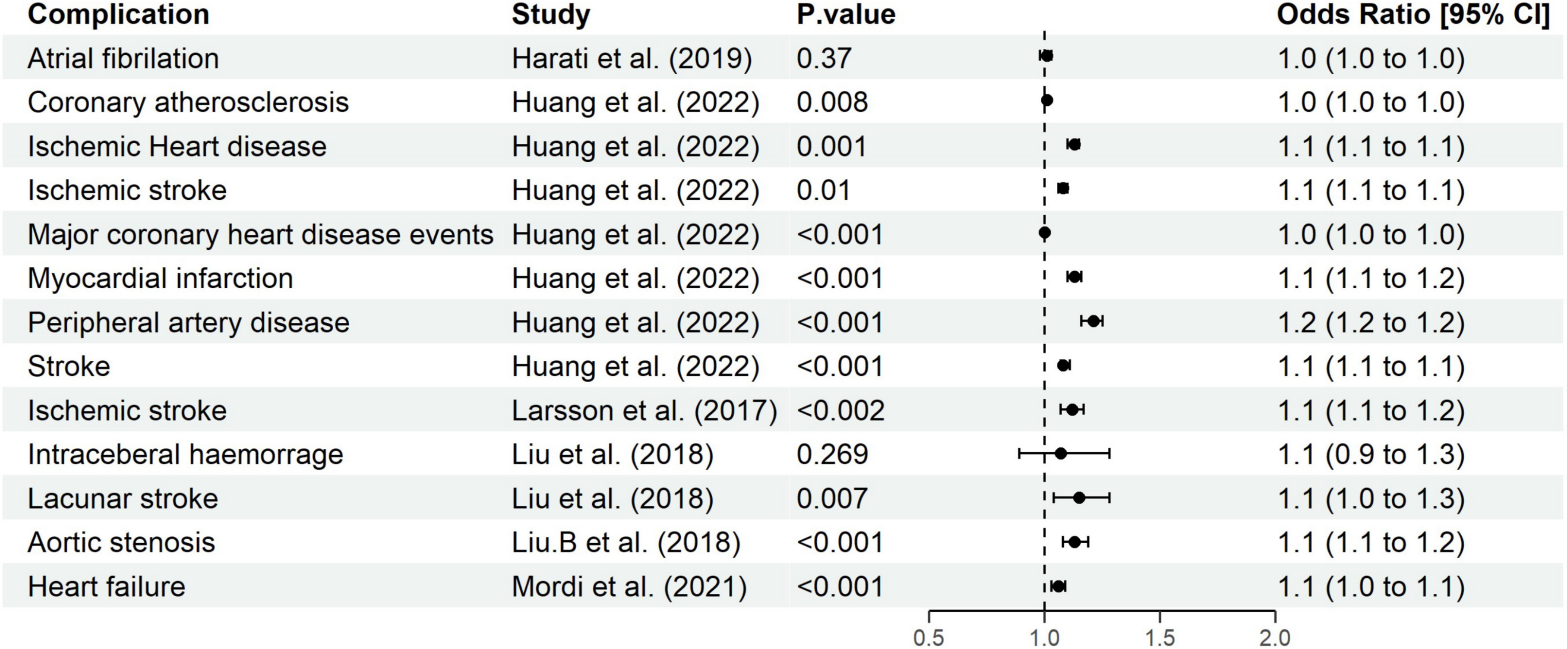
Type 2 diabetes causal effect on micro/macrovascular complications. This forest plot shows the result of several Mendelian randomization studies using genetic variants associated with type 2 diabetes as instrumental variables to investigate whether genetically predicted type 2 diabetes increases the risk of developing microvascular and macrovascular complications. Results shows the corresponding change in risk expressed in odds ratio (OR, 95% CI) on the *x‐*axis for genetically increased risk of type 2 diabetes, while the *y*‐axis shows different microvascular and macrovascular complications.

Several Mendelian randomization studies have been conducted to identify a causal association between type 2 diabetes and its associated macro‐ and microvascular complications. These studies suggest that genetically predicted higher risk of type 2 diabetes is associated with higher risk of coronary atherosclerosis, ischaemic heart disease, ischaemic stroke, myocardial infarction, peripheral artery disease, aortic valve stenosis and heart failure using data from mainly European ancestries^26^, ^27^, ^28^ (Figure 4). The genetic evidence does not support a causal role of type 2 diabetes on risk of atrial fibrillation or intracerebral haemorrhage.^28^, ^29^

## 6 WHAT ASPECT OF TYPE 2 DIABETES IS DRIVING THE RISK?

Type 2 diabetes is a collection of different metabolic features and events which make it a complex heterogeneous disease in terms of clinical presentation, disease course, response to treatment and complication risk. These metabolic features include β‐cell dysfunction, insulin resistance, lipodystrophy (in small number of cases), excess adiposity and lipid pathways.^30^ The contribution of the metabolic derangements to the development of type 2 diabetes can be markedly different among affected individuals. Evidence offered by genetic studies has introduced the concept of subtypes of type 2 diabetes^30^, ^31^, ^32^ although this area remains highly controversial and contested.^33^, ^34^

Any association between type 2 diabetes and vascular complications could be driven by a particular metabolic trait or pathways. With personalized and precision medicine in type 2 diabetes rapidly evolving, understanding which type 2 diabetes components are mostly associated with the risk of micro‐ and macrovascular complications could provide an opportunity for individualized treatment and management plan. This insight will allow clinicians to predict the risk of specific vascular complications based on the associated diabetes component and provide a management plan consequently. For example, if excess adiposity is causally associated with risk of diabetic kidney disease, people with type 2 diabetes with higher BMI levels should receive medications that lower weight more for potentially greater protection. This remains speculative and trials are needed to provide more evidence.

In the following section, we discuss how hyperglycaemia, insulin resistance, obesity and hypertension could influence risk of vascular complications using evidence from Mendelian randomization studies (Table 1).

**TABLE 1:** Summary of Mendelian randomization studies examining whether type 2 diabetes or its associated components are causally associated with higher risk of micro‐ and macrovascular diseases. The table include the following columns

Exposure	Study	Complication	OR	LCI	UCI	n.Case	n.Contr	Cohort	*p* value	Unit	Disease definition	Pleiotropy	n.SNPs
BMI	Chatterjee et al. (2017)	Atrial fibrillation	1.11	1.05	1.18	4178	51,646	AGES/ARIC/FHS/PREVEND/WGHS	<0.001	1‐SD increase in BMI	Cases: AF diagnosed on ECG, Holter, or obtained from clinicians records, Controls: healthy	Final result after adjusting for pleiotropic SNPs	NA
BMI	Larsson et al. (2020)	Abdominal aortic aneurysm	1.06	0.96	1.16	758	3,66,945	UKBB	0.25	1‐kg/m^2^ increase in BMI	Cases: aortic aneurysm, controls: Healthy	Final result consistent with sensitivity analysis	96
BMI	van't Hof et al. (2017)	Abdominal aortic aneurysm	1.63	0.99	2.61	818	3004	Dutch population	<0.002	1‐SD increase in BMI	Cases: ruptured/non‐ruptured intracranial and abdominal Aortic aneurysm, controls: healthy	Final result consistent with sensitivity analysis	97
BMI	Larsson et al. (2020)	Coronary artery disease	1.07	1.04	1.09	24,531	3,43,172	UKBB	1.30 E‐05	1‐kg/m^2^ increase in BMI	Cases versus healthy controls	Final result consistent with sensitivity analysis	404
BMI	Larsson et al. (2020)	Ischaemic stroke	1.03	0.99	1.07	3554	3,64,149	UKBB	<0.001	1 kg/m^2^ higher BMI	Cases: ischaemic stroke, controls: HeBurgessthy	Final result consistent with sensitivity analysis	96
BMI	Larsson et al. (2020)	Peripheral artery disease	1.66	1.56	1.76	3514	3,64,189	UKBB	1.40 E‐03	1 kg/m^2^ increase in BMI	Cases: peripheral artery disease, controls: HeBurgessthy	Final result consistent with sensitivity analysis	96
BMI	Todd et al. (2015)	Diabetic kidney disease	1.33	1.17	1.51	2916	3315	UK‐ROI/FinnDiane/GoKinD US	0.62	1 kg/m^2^ higher BMI	Cases: T1D with ACR >300 and ESRD, cases with ESRD, cases. Controls: T1D	Final result after adjusting for pleiotropic SNPs	32
BMI	Todd et al. (2015)	End‐stage renal disease	1.43	1.2	1.72	2916	3315	UK‐ROI/FinnDiane/GoKinD US	<0.001	1 kg/m^2^ higher BMI	T1D with Macroalbuminuria cases versus T1D without ESRD, controls T1D	Final result after adjusting for pleiotropic SNPs	32
BMI	Todd et al. (2015)	Macroalbumiuria	1.28	1.11	1.45	2916	3315	UK‐ROI/FinnDiane/GoKinD US	0.001	1 kg/m^2^ higher BMI	Cases: T1D with ACR >300, Controls: T1D	Final result after adjusting for pleiotropic SNPs	32
BMI	Zheng et al. (2021)	Chronic kidney disease	1.78	1.64	1.94	51,672	9,58,102	CKDGen, UK Biobank and HUNT	<0.001	1‐SD increase in BMI	CKD cases versus controls	Final result consistent with sensitivity analysis	902
BMI	Huang et al. (2016)	Peripheral artery disease	1.44	1.18	1.75	707	10,776	Chinese population from Shanghai	0.0004	1‐SD increase in BMI GRS	Cases: having ABI <0.9 or >1.4 at either side, controls: Healthy	Final result consistent with sensitivity analysis	14
BMI	Martin et al. (2022)	Coronary artery disease	1.41	1.1	1.81	43,054	4,07,969	CIHDS/CCHS/CGPS/ Cardiogram	0.007	1‐SD increase in BMI	cases: IHD/stenosis/atherosclerosis/positive ECG/ MI, controls: Healthy, controls: healthy	Final result consistent with sensitivity analysis	73
BMI	Martin et al. (2022)	Stroke	1.19	1.07	1.31	14,171	1,33,027	Finngen/Published GWAS	1.00 E‐03	1‐SD increase in BMI	Cases: stroke as per WHO definition, controls: Healthy	Final result consistent with sensitivity analysis	73
BMI	Martin et al. (2022)	Peripheral artery disease	1.87	1.46	2.39	5323	1,67,843	Chinese population	4.00 E‐06	1‐SD increase in BMI	11,837 Chinese participants from Shanghai	Final result consistent with sensitivity analysis	73
BMI	Martin et al. (2022)	Atrial fibrillation	1.65	1.33	2.05	17,325	97,214	Finngen/Published GWAS	5.00 E‐06	1‐SD increase in BMI	Cases: Paroxysmal or permanent atrial fibrillation, or atrial flutter, controls: Healthy	Final result consistent with sensitivity analysis	73
BMI	Shah et al. (2020)	Heart failure	1.61	1.45	1.79	47,309	9,30,014	HERMES, UK Biobank	2.70 E‐50	1‐SD increase in BMI	Cases: Heart failure, controls: Healthy	Final result consistent with sensitivity analysis	89
BMI	Martin et al. (2022)	Heart failure	1.86	1.6	2.16	9576	1,59,286	Finngen/Published GWAS	2.00 E‐16	1‐SD increase in BMI	Cases: any aetiology of heart failure with no inclusion criteria based on left ventricular ejection fraction, Controls: Healthy	Final result consistent with sensitivity analysis	73
BMI	Martin et al. (2022)	Chronic kidney disease	1.21	1.08	1.36	2821	1,72,745	Finngen	0.002	1‐SD increase in BMI	Cases: eGFR<60?ml?min–1?per 1.73?m2, controls: Healthy	Final result consistent with sensitivity analysis	73
BMI	Martin et al. (2022)	Abdominal aortic aneurysm	1.16	0.83	1.62	1919	1,67,843	Finngen/Published GWAS	0.394	1‐SD increase in BMI	Cases: an infrarenal aortic diameter greater than 30 mm excluding secondary aneurysm, Controls: Healthy	Final result consistent with sensitivity analysis	73
DBP	Wan et al. (2021)	Cardiovascular disease	1.05	0.96	1.16	45,746	50,216	UKBB, European British population	0.6	5 mm/Hg DBP increase	Cases: defined according to ICD‐9 and ICD‐10 codes, Controls: Healthy	Final result consistent with sensitivity analysis	364
DBP	Wan et al. (2021)	Ischaemic heart disease	1.04	0.93	1.16	36,748	50,216	UKBB, European British population	0.7	5 mm/Hg DBP increase	Cases: defined according to ICD‐9 and ICD‐10 codes, Controls: Healthy	Final result consistent with sensitivity analysis	364
DBP	Wan et al. (2021)	Myocardial infarction	1.09	0.96	1.24	27,500	50,216	UKBB, European British population	0.4	5 mm/Hg DBP increase	Cases: defined according to ICD‐9 and ICD‐10 codes, Controls: Healthy	Final result consistent with sensitivity analysis	364
DBP	Wan et al. (2021)	Stable angina	1.16	0.98	1.28	21,119	50,216	UKBB, European British population	0.4	5 mm/Hg DBP increase	Cases: defined according to ICD‐9 and ICD‐10 codes, Controls: Healthy	Final result consistent with sensitivity analysis	364
DBP	Wan et al. (2021)	Unstable angina	1.12	0.66	1.02	6190	50,216	UKBB, European British population	0.4	5 mm/Hg DBP increase	Cases: defined according to ICD‐9 and ICD‐10 codes, Controls: Healthy	Final result consistent with sensitivity analysis	364
DBP	Wan et al. (2021)	Stroke	0.82	0.71	0.96	10,785	50,216	UKBB, European British population	0.2	5 mm/Hg DBP increase	Cases: defined according to ICD‐9 and ICD‐10 codes, Controls: Healthy	Final result consistent with sensitivity analysis	364
DBP	Wan et al. (2021)	Ischaemic stroke	0.9	0.76	1.06	9165	50,216	UKBB, European British population	0.5	5 mm/Hg DBP increase	Cases: defined according to ICD‐9 and ICD‐10 codes, Controls: Healthy	Final result consistent with sensitivity analysis	364
DBP	Wan et al. (2021)	Intracerebral haemorrhage	0.64	0.41	1	1154	50,216	UKBB, European British population	0.3	5 mm/Hg DBP increase	Cases: defined according to ICD‐9 and ICD‐10 codes, Controls: Healthy	Final result consistent with sensitivity analysis	364
DBP	Wan et al. (2021)	Heart failure	0.93	0.78	1.11	7650	50,216	UKBB, European British population	0.7	5 mm/Hg DBP increase	Cases: defined according to ICD‐9 and ICD‐10 codes, Controls: Healthy	Final result consistent with sensitivity analysis	364
DBP	Wan et al. (2021)	Peripheral vascular disease	0.83	0.69	1	8131	50,216	UKBB, European British population	0.3	5 mm/Hg DBP increase	Cases: defined according to ICD‐9 and ICD‐10 codes, Controls: Healthy	Final result consistent with sensitivity analysis	364
DBP	Wan et al. (2021)	Arrhythmia	0.9	0.75	1.09	24,637	50,216	UKBB, European British population	0.6	5 mm/Hg DBP increase	Cases: defined according to ICD‐9 and ICD‐10 codes, Controls: Healthy	Final result consistent with sensitivity analysis	364
Fasting glucose	Ahmad et al. (2015)	Coronary Heart disease	1.15	1	1.32	63,746	1,30,681	Cardiogramplus	0.05	0.025 mmol/L per allele	Cases: non‐diabetic participants with CHD, controls: healthy	Final result after excluding pleiotropic SNPs	24
Fasting glucose	Harati et al. (2019)	Atrial fibrillation	0.95	0.82	1.09	60,620	9,70,210	HUNT/DECODE/MGI/DiscovEHR/ AFGen	0.49	1‐SD increase/mmol	Cases: AF, controls: Healthy	Final result after excluding pleiotropic SNPs	36
Fasting glucose	Kim et al. (2020)	Chronic kidney disease	0.99	0.98	1	5909	10,030	Korean population/KoGES/KARE	0.098	1‐SD increase/mmol	General population	Final result after excluding pleiotropic SNPs	9
Fasting glucose	Merino et al. (2017)	Coronary artery disease	1.43	1.14	1.79	63,746	1,30,681	UKBB	0.02	1‐mmol/L increase in FG	Cases: participants with CAD, controls: healthy	Final result after excluding pleiotropic SNPs	11
Fasting glucose	Ross et al. (2015)	Coronary heart disease	1.18	0.97	1.42	85,979	1,95,443	Cardiogramplus	>0.05	No causal effect	Cases: participants with CAD, controls: healthy	Final result after excluding pleiotropic SNPs	30
Fasting Insulin	Larsson et al. (2017)	Ischaemic stroke	1.03	0.78	1.37	37,296	18,476	METASTROKE, NINDS‐SiGN	0.82	1‐SD increase for FI	Cases: ischaemic stroke, controls: Healthy	Final result consistent with sensitivity analysis	18
Fasting Insulin	Liu et al. (2018)	Intracerebral haemorrhage	0.48	0.12	1.86	2191	27,297	NA	0.288	NA	Cases: intracerebral haemorrhage, controls: Healthy matched for age, sex, race	Final result after excluding pleiotropic SNPs	9
Fasting Insulin	Liu et al. (2018)	Lacunar stroke	1.52	0.45	5.08	2191	27,297	NA	0.5	NA	Cases: lacunar stroke, controls: Healthy	Final result after excluding pleiotropic SNPs	9
Fasting Insulin	Tikkanen et al. (2016)	Coronary heart disease	1.06	1.02	1.1	5834	11,668	FINRISK/DILGOM/Corogene/Genmets	0.002	1‐SD increase of GRS	Cases: MI, unstable angina/coronary revasc, death from CHD, controls: healthy	Final result after excluding pleiotropic SNPs	20
Fasting Insulin	Zhan et al. (2017)	Coronary Heart disease	1.86	1.01	3.41	22,233	64,762	CARDIoGRAMplusC4D/ENGAGE	0.04	log‐transformed fasting insulin	Cases: coronary heart disease, controls: Healthy	Final result after excluding pleiotropic SNPs	10
HbA_1c_	Harati et al. (2019)	Atrial fibrillation	1.01	0.85	1.17	60,620	970,21	HUNT/DECODE/MGI/DiscovEHR/ AFGen	0.88	1‐SD mol (%) for HbA_1c_	Cases: AF, controls: Healthy	Final result after excluding pleiotropic SNPs	11
HbA_1c_	Leong et al. (2019)	Coronary heart disease	1.61	1.4	1.84	79,716	5,79,475	UKBB, Cardiogramplus	1.00 E‐09	1‐SD increase in BMI	Cases: coronary heart disease, controls: Healthy	Final result suggests presence of pleiotropy	36
HbA_1c_	Mutie et al. (2020)	Coronary artery disease	1.03	0.64	1.64	1,23,733	4,24,528	UKBB, Cardiogramplus	>0.05	No causal effect	Cases: coronary artery disease, controls: Healthy	Final result consistent with sensitivity analysis	10
HbA_1c_	Ross et al. (2015)	Coronary artery disease	1.53	1.14	2.05	85,979	1,95,443	UKBB, Cardiogramplus	0.002	1% increase in HbA_1c_	Cases: coronary artery disease, controls: Healthy	Final result after excluding pleiotropic SNPs	9
Insulin Resistance (IR)	Chen et al. (2020)	Coronary artery disease	1.79	1.57	2.04	60,801	1,23,504	GLGC, CARDIOGRAM/GENESIS	<0.001	1‐SD increase in IR	Cases: ischaemic stroke/stroke subtypes, controls: Healthy	Final result consistent with sensitivity analysis	52
Insulin Resistance (IR)	Chen et al. (2020)	Ischaemic stroke	1.21	1.05	1.4	67,162	4,54,450	GLGC, CARDIOGRAM/GENESIS	0.007	1‐SD increase in IR	Cases: ischaemic stroke/stroke subtypes, controls: Healthy	Final result consistent with sensitivity analysis	52
Insulin Resistance (IR)	Chen et al. (2020)	Myocardial infarction	1.78	1.54	2.06	60,801	1,23,504	GLGC, CARDIOGRAM/GENESIS	<0.001	1‐SD increase in IR	Cases: ischaemic stroke/stroke subtypes, controls: Healthy	Final result consistent with sensitivity analysis	52
Insulin Resistance (IR)	Chen et al. (2020)	Small‐artery occlusion type stroke	1.8	1.3	2.49	67,162	4,54,450	GLGC, CARDIOGRAM/GENESIS	<0.001	1‐SD increase in IR	Cases: ischaemic stroke/stroke subtypes, controls: Healthy	Final result consistent with sensitivity analysis	52
Insulin Resistance (IR)	Zhao et al. (2019)	Atrial fibrillation	3.23	1.88	5.56	14,442	3,92,010	UKBB	0.004	1‐SD increase in IR	Cases: atrial fibrillation, controls: Healthy	Final result after excluding pleiotropic SNPs	7
Non‐fasting glucose	Benn et al. (2012)	Ischaemic Heart disease	1.25	1.03	1.52	14,155	66,367	CIHDS/CCHS/CGPS	<0.001	1‐mmol/L increase NFBG	Cases: IHD/stenosis/atherosclerosis/positive ECG/ MI, controls: Healthy	Final result consistent with sensitivity analysis	5
Non‐fasting glucose	Benn et al. (2012)	Myocardial infarction	1.69	1.28	2.23	6257	74,265	CIHDS/CCHS/CGPS	<0.001	1‐mmol/L increase NFBG	Cases: IHD/stenosis/atherosclerosis/positive ECG/ MI, controls: Healthy	Final result consistent with sensitivity analysis	5
SBP	Wan et al. (2021)	Unstable angina	1.69	1.38	2.08	6190	50,216	UKBB, European British population	0.01	10 mm/Hg SBP increase	Cases: defined according to ICD‐9 and ICD‐10 codes, Controls: Healthy	Final result consistent with sensitivity analysis	327
SBP	Wan et al. (2021)	Stroke	1.72	1.49	2	10,785	50,216	UKBB, European British population	0.0003	10 mm/Hg SBP increase	Cases: defined according to ICD‐9 and ICD‐10 codes, Controls: Healthy	Final result consistent with sensitivity analysis	327
SBP	Wan et al. (2021)	Ischaemic stroke	1.55	1.32	1.82	9165	50,216	UKBB, European British population	0.007	10 mm/Hg SBP increase	Cases: defined according to ICD‐9 and ICD‐10 codes, Controls: Healthy	Final result consistent with sensitivity analysis	327
SBP	Wan et al. (2021)	Intracerebral haemorrhage	2.57	1.66	3.97	1154	50,216	UKBB, European British population	0.03	10 mm/Hg SBP increase	Cases: defined according to ICD‐9 and ICD‐10 codes, Controls: Healthy	Final result consistent with sensitivity analysis	327
SBP	Wan et al. (2021)	Heart failure	1.42	1.2	1.69	7650	50,216	UKBB, European British population	0.04	10 mm/Hg SBP increase	Cases: defined according to ICD‐9 and ICD‐10 codes, Controls: Healthy	Final result consistent with sensitivity analysis	327
SBP	Wan et al. (2021)	Peripheral vascular disease	1.39	1.16	1.66	8131	50,216	UKBB, European British population	0.04	10 mm/Hg SBP increase	Cases: defined according to ICD‐9 and ICD‐10 codes, Controls: Healthy	Final result consistent with sensitivity analysis	327
SBP	Wan et al. (2021)	Arrhythmia	1.32	1.18	1.7	24,637	50,216	UKBB, European British population	0.06	10 mm/Hg SBP increase	Cases: defined according to ICD‐9 and ICD‐10 codes, Controls: Healthy	Final result consistent with sensitivity analysis	327
Type 2 diabetes	Harati et al. (2019)	Atrial fibrillation	1.01	0.98	1.03	60,620	970,21	HUNT/DECODE/MGI/DiscovEHR/AFGen	0.37	1‐SD increase/mmol	Cases: AF, controls: Healthy	Final result after excluding pleiotropic SNPs	122
Type 2 diabetes	Huang et al. (2022)	Coronary atherosclerosis	1.01	1	1.01	60,801	1,23,504	UKBB	0.008	1‐SD increase mmol/mol	Cases: coronary atherosclerosis, controls: Healthy	Final result consistent with sensitivity analysis	277
Type 2 diabetes	Huang et al. (2022)	Ischaemic Heart disease	1.13	1.1	1.15	60,801	1,23,504	CARDIoGRAMplusC4D	0.001	Genetically increased T2D	Cases: ischaemic heart disease, controls: Healthy	Final result consistent with sensitivity analysis	269
Type 2 diabetes	Huang et al. (2022)	Ischaemic stroke	1.08	1.06	1.1	67,162	4,54,450	MEGASTROKE	0.01	Genetically increased T2D	Cases: ischaemic stroke, controls: Healthy	Final result consistent with sensitivity analysis	269
Type 2 diabetes	Huang et al. (2022)	Major coronary heart disease events	1	1.002	1.004	60,801	1,23,504	UKBB	<0.001	Genetically increased T2D	Cases: major coronary heart disease events, controls: Healthy	Final result consistent with sensitivity analysis	231
Type 2 diabetes	Huang et al. (2022)	Myocardial infarction	1.13	1.1	1.16	60,801	1,23,504	CARDIoGRAMplusC4D	<0.001	Genetically increased T2D	Cases: myocardial infarction, controls: Healthy	Final result consistent with sensitivity analysis	231
Type 2 diabetes	Huang et al. (2022)	Peripheral artery disease	1.21	1.16	1.25	60,801	1,23,504	CARDIoGRAMplusC4D	<0.001	Genetically increased T2D	Cases: peripheral artery disease, controls: Healthy	Final result consistent with sensitivity analysis	217
Type 2 diabetes	Huang et al. (2022)	Stroke	1.08	1.07	1.11	67,162	4,54,450	MEGASTROKE	<0.001	Genetically increased T2D	Cases: stroke, controls: Healthy	Final result consistent with sensitivity analysis	231
Type 2 diabetes	Larsson et al. (2017)	Ischaemic stroke	1.12	1.07	1.17	18,476	37,296	MEGASTROKE/NINDS/SiGN	3.00 E‐06	1‐unit‐higher log‐odds for T2D	Cases: ischaemic stroke, controls: Healthy	Final result consistent with sensitivity analysis	49
Type 2 diabetes	Liu et al. (2018)	Intracerebral haemorrhage	1.07	0.89	1.28	2254	8195	CDK portal/Cambridge ICH/UKBB	0.269	No causal effect	Cases: intracerebral haemorrhage, controls: Healthy	Final result after excluding pleiotropic SNPs	77
Type 2 diabetes	Liu et al. (2018)	Lacunar stroke	1.15	1.04	1.28	2191	27,297	CDK portal/Cambridge ICH/UKBB	0.007	Twofold increase in T2D	Cases: lacunar stroke, controls: Healthy	Final result after excluding pleiotropic SNPs	77
Type 2 diabetes	Liu et al. (2018)	Aortic stenosis	1.13	1.08	1.19	2244	3,67,703	UKBB	<0.001	Twofold increase in T2D	Cases: patients with aortic valve stenosis according to ICD9, ICD10, Controls: Healthy	Final result after excluding pleiotropic SNPs	243
Type 2 diabetes	Mordi et al. (2021)	Heart failure	1.06	1.03	1.09	47,309	9,30,014	HERMES, European population	<0.001	1‐log unit higher odds of T2D	Cases: physician diagnosis of HF, image diagnosis, ICD codes, Controls: Healthy	Final result suggests presence of pleiotropy	763

*Note*: Exposure: The genetic instrument used for the respective Mendelian randomization study. Study: Author and year of publication. Complication: the outcome studied. OR (odds ratio), LCI (lower confidence interval) and UCI (upper confidence interval): change in the risk of outcome. n.Case (number of cases) and n.Contr (number of controls). Cohort: information about the ethnic group of each study. *p* value: the statistical significance of the exposure vs outcome association. Unit: for the effect size of each exposure. Disease definition: Definition of each outcome/complication. Pleiotropy: indicates either horizontal pleiotropy present, pleiotropic SNPs were removed, or no pleiotropy detected. n.SNPs: the number of SNPs identified to be associated with outcome in each study.

### 6.1 Hyperglycaemia

Both observational and genetic studies have investigated the role of hyperglycaemia in developing diabetes‐associated micro‐ and macrovascular complications. However, the mechanism by which the risk for those complications is driven remains vague, notably in diabetic peripheral neuropathy.^35^ Mendelian randomization using genetic variants associated with hyperglycaemia started in 2015. Studies investigated the impact of hyperglycaemia from the prediabetes stage to understand the glycaemic association with vascular complications. In this context, 47 genetic variants from the Meta‐Analyses of Glucose and Insulin‐related traits Consortium (MAGIC) associated with fasting blood glucose in nondiabetic range were used to investigate the causal inference of prediabetes on coronary artery disease, stroke and chronic kidney disease. The results of this study suggested that 1 mmol/L higher fasting blood glucose in individuals without diabetes increased the risk of coronary artery disease by an odds ratio of 1.26 (95% confidence interval [CI]: 1.16, 1.38) and concluded that high fasting glucose in prediabetes is only causally associated with coronary artery disease but not stroke or chronic kidney disease.^36^ Another study investigated the causal inference of higher HbA_1c_ on the increased risk for cardiovascular diseases, namely haemorrhagic stroke, peripheral vascular disease and pulmonary embolism and found genetically predicted higher HbA_1c_ was associated with higher risk of coronary artery disease and stroke.^37^

A recent comprehensive Mendelian randomization study was conducted by Emanuelsson et al^38^ to investigate whether high non‐fasting glucose levels in the normoglycaemic range (individuals with non‐fasting glucose 2 h after meal ≥4. to 11.0 mmol/L) and below the diabetes cut‐off point (11.1 mmol/L) are causally associated with an increased risk of retinopathy, neuropathy, nephropathy, chronic kidney disease, peripheral arterial disease and myocardial infarction. They used genetic variants associated with high blood glucose among non‐diabetic people (including *GCP62/ABCB1* (rs560887), *GCK* (rs4607517), *DGKB* (rs2191349), *ADCY5* (rs11708067), *CDKN2A/B* (rs10811661 and rs2383206) and *TCF7L2* (rs7903146)) and found that 1 mmol/L higher non‐fasting glucose in the normoglycaemic range is associated with higher risk of retinopathy (risk ratio 2.01 [95% CI 1.18–3.41]), peripheral neuropathy (2.15 [1.38–3.35]), diabetic nephropathy (1.58 [1.04–2.40]) and peripheral artery disease (1.19 [0.90–1.58]). While this is interesting, an important limitation of that study is the lack of fasting glucose or HbA_1c_ data and therefore it is difficult to be certain of the validity of these findings generated on random glucose levels.

### 6.2 Insulin resistance

A study investigated the causal association between 53 genetic variants associated with insulin resistance (variants associated with elevated fasting insulin, lower HDL‐C and higher triglyceride levels) reported a significant higher risk of coronary heart disease in general population after adjusting for fasting insulin and BMI (odds ratio 1.79, 95% CI: [1.57–2.04], *p* < 0.001), ischaemic stroke (1.21 [1.05–1.40], *p* = 0.007), small‐artery occlusion subtype of stroke (1.80 [1.30–2.49], *p* < 0.001) and myocardial infarction (1.78 [1.54–2.06], *p* < 0.001) per 1‐SD (standard deviation) increase in insulin resistance phenotype for all outcomes.^39^ Even though observational studies have linked insulin resistance to microvascular complications, the shortcomings of observational studies in terms of bias and reverse causation emphasize the need for further investigations to establish a causal association between insulin resistance and microvascular complications.

### 6.3 Body mass index

Body mass index (BMI), as a measure of obesity, has been the most studied risk factor. The association between obesity and macrovascular complications has been reported by observational studies.^40^, ^41^ However, despite the similarity in aetiology between macro‐ and microvascular complications, observational studies have been inconsistent in establishing an association between obesity and microvascular complications. Several relevant Mendelian randomization studies have been published. For instance, 1‐SD increase in genetically estimated BMI was associated with a higher risk of diabetic nephropathy (odds ratio 3.76, 95% CI [1.88–7.53], *p* < 0.001) and reduced eGFR levels (estimated glomerular filtration rate) (0.71, [0.59–0.86], *p* < 0.001); however, no association was found between BMI and proteinuria.^42^ Another study on the causal effect of childhood BMI on the risk of adult type 2 diabetes, coronary artery disease and nephropathy using 15 genetic variants identified by the Early Growth Genetics (EGG) consortium found that a 1‐SD increase in childhood BMI was significantly associated with an increased risk of the adult onset of type 2 diabetes ranging from 47% to 83% (odds ratio 1.47 [1.18, 1.82] to 1.83 [1.46, 2.30]), 28% increased risk of adult coronary arteries disease (1.28 [1.17, 1.39]), but a borderline association was found with adult chronic kidney disease (1.14 [0.99, 1.31]).^43^ Another study used 97 genetic variants associated with BMI from the GIANT consortium (The Genetic Investigation of ANthropometric Traits) to assess the causal association between obesity and various human diseases. Contrary to the findings of some observational studies, which reported a lower risk of diabetic retinopathy to be associated with a higher BMI,^44^ this study found that genetically elevated BMI was an independent causal risk factor for diabetic retinopathy. This study once again suggests that results from observational studies which examine risks linked to BMI, may be misleading due to the unintentional loss of weight associated with long‐standing diabetes.^45^

## 7 THE ROLE OF HYPERTENSION IN DIABETES‐ASSOCIATED MICRO‐ AND MACROVASCULAR COMPLICATIONS

Diabetes and hypertension have several pathophysiological links due to common risk factors and complications. Macrovascular complications, for instance, are common findings in people with diabetes, hypertension or both. On the other hand, microvascular complications such as nephropathy, neuropathy and retinopathy are thought to be accelerated by hypertension.^46^

Individuals diagnosed with type 2 diabetes are found to have a twofold risk of hypertension compared to healthy individuals, while those diagnosed with hypertension often exhibit insulin resistance and are at a higher risk of developing type 2 diabetes. Risk factors in the form of alcohol consumption, unhealthy lifestyle and obesity are behind the development of both conditions.^47^ The prevalence of coexistence of diabetes and hypertension ranges from 19% to 51% in Asian and Western countries. The UK Prospective Diabetes Study of systolic blood pressure among people with diabetes and any incident of microvascular or macrovascular complications reported a hazard ratio of 1.12 (*p* < 0.001) per 10 mm Hg increments of systolic blood pressure. The study also found that individuals with HbA_1c_ ⩾64 mmol/mol and systolic blood pressure ⩾150 mm Hg had a 16.3‐fold higher risk of developing microvascular complications than those with HbA_1c_ < 42 mmol/mol and systolic blood pressure <130 mm Hg.^48^

Type 2 diabetes has been found to be associated with a higher risk of hypertension and vice versa; however, the causality between both conditions remains uncertain. A bidirectional Mendelian randomization study was conducted on participants from the UK biobank study using genetic variants for type 2 diabetes and hypertension. The study found that type 2 diabetes is causally associated with higher risk of hypertension (odds ratio 1.07 [95% CI, 1.04–1.10], *p* = 3.4 × 10^−7^), while no causal link was detected for hypertension causing type 2 diabetes (odds ratio 0.96 [0.88–1.04], *p* = 0.34). Moreover, type 2 diabetes was associated with 0.67 mm Hg higher systolic blood pressure (95% CI 0.41–0.93, *p* = 5.75 × 10^−7^), but no association was seen with diastolic blood pressure.^49^ Mendelian randomization studies to investigate the role of hypertension in risk of diabetes vascular complications are mainly limited to macrovascular conditions and have provided evidence for a causal role of hypertension in higher risk of cardiovascular disease, stroke, myocardial infarction, heart failure and peripheral vascular disease. A recent study used 327 and 364 genetic variants strongly and independently associated with systolic and diastolic blood pressure, respectively, found that 10 mm Hg increase in systolic blood pressure was associated with increased risk of total cardiovascular disease (odds ratio 1.32 [95% CI, 1.25–1.40]), ischaemic heart disease (1.33, [1.24–1.41]) and stroke (1.35, [1.24–1.48]), while 5 mm Hg increase in diastolic blood pressure was causally associated with total cardiovascular disease (1.20 [1.14–1.27]), ischaemic heart disease (1.20 [1.15–1.26]) and stroke (1.20 [1.12–1.28]).^50^

## 8 EVIDENCE FROM STUDIES OF NON‐EUROPEANS

Mendelian randomization studies in non‐European are scarce due to limited genome‐wide association study (GWAS) data. Few studies investigated the causal effect of type 2 diabetes and its components on the risk of vascular complications. A Mendelian randomization study by Jie Zheng et al.^51^ investigated the causal effect of 45 cardiometabolic risk factors, including type 2 diabetes, on the risk of chronic kidney disease among European and three East Asian Biobanks. The study found that type 2 diabetes causally increased the risk of chronic kidney disease among all three populations (Europeans, Chinese and Japanese) consistently. BMI increased the risk of chronic kidney disease among Europeans and individuals from the Japanese Biobanks but not among the China Kadoorie individuals which could be due to either limited cases of chronic kidney disease in the China Kadoorie Biobank or ethnic‐based difference. Systolic blood pressure had strong causal effect among Europeans but showed no evidence among East Asian population, which could indicate an ancestry‐based role for systolic blood pressure in the development of chronic kidney disease. Another Mendelian randomization study by Xuehao et al.^52^ found consistent causal effect of type 2 diabetes on higher risk of peripheral artery disease among both European and East Asian individuals. Such evidence could suggest that type 2 diabetes is less likely to be affected by ethnic variation in the development of vascular complications, however, multiple studies among different ethnic groups are needed for better judgement and understanding.

## 9 CONCLUSION AND FUTURE WORK

Based on the available evidence from both observational and genetic studies, there appears a causal role played by type 2 diabetes in the development of cardiovascular disease, coronary heart disease, stroke, retinopathy and nephropathy but less clear in diabetic neuropathy and other macrovascular complications such as intracerebral haemorrhage and peripheral artery disease.

Given that type 2 diabetes is a disease of ectopic fat mass (including in liver and circulation as higher triglycerides), and that high BMI is one of its main risk factors, it is notable that higher BMI appear causal for not only cardiovascular complication, but also diabetic nephropathy, low eGFR as well as retinopathy. Type 2 diabetes and higher BMI also appear causal for hypertension, and it is important to note hyperglycaemia is also related to cardiovascular complications. Clearly, much more genetic work needs to be done to tease out to what extent each of the type 2 diabetes risk factors (or its underlying pathways) are relevant for differing complications but the data to date, as summarized, suggest an important role for excess weight in range of type 2 diabetes complications. This is interesting, as there appears to be an increasing focus on treating excess weight in the management of type 2 diabetes, both for remission^53^ and potentially reduction in multiple complications.^54^ This focus on a need to target excess weight more in type 2 diabetes has also been recognized in the recently updated ADA/EASD recommendations.^55^ The work summarized also provide more evidence for a multifactorial approach (targeting not only glycaemia but also blood pressure, excess weight and lipids) to treating type 2 diabetes to prevent complications. Further developments in genetic analyses should help tease out relative contributions of each diabetes component on its various complications, findings which could translate to better defined intervention trials and, eventually, to clinical guidelines.

## CONFLICT OF INTEREST

NS has received grant and personal fees from AstraZeneca, Boehringer Ingelheim and Novartis; grant from Roche Diagnostics; and personal fees from Abbott Laboratories, Afimmune, Amgen, Eli Lilly, Hanmi Pharmaceuticals, Merck Sharp & Dohme, Novo Nordisk, Pfizer and Sanofi outside the submitted work.

## Data Availability

I confirm that my Data Availability Statement (pasted below) complies with the Expects Data Policy. The data that support the findings of this study are available in the supplementary material of this article.
